# Kinematic analysis of posterior-stabilized total knee arthroplasty during standing up from and sitting down on a chair

**DOI:** 10.1186/s13018-016-0482-y

**Published:** 2016-11-17

**Authors:** Takatomo Mine, Kenji Hoshi, Kazuyoshi Gamada, Koichiro Ihara, Hiroyuki Kawamura, Ryutaro Kuriyama, Ryo Date

**Affiliations:** 1 Department of Orthopaedic Surgery, National Hospital Organization Kanmon medical Center, 1-1 ChofuUshiroda Simonoseki, Yamaguchi, 752-8510 Japan; 2 Department of Rehabilitation, Hiroshima International University, Hiroshima, Japan

**Keywords:** Kinematics, Standing up from and sitting down on a chair motion, Total knee arthroplasty, Posterior stabilized total knee arthroplasty, Post-cam mechanism

## Abstract

**Background:**

Total knee arthroplasty is effective to regain quality of life. Standing up from and sitting down on a chair and stair stepping motion are important in daily living. We previously reported in vivo kinematics of this implant during a stepping exercise. The purpose of this analysis was to assess in vivo knee motion during standing up from and sitting down on a chair and determine the motion pattern in patients with the unique knee prosthesis.

**Methods:**

A total of 15 patients implanted with Bi-Surface PS were assessed during standing up from and sitting down on a chair. The Bi-Surface PS knee is a posterior-cruciate substitute prosthesis with a unique ball-and-socket joint in the mid-posterior portion of the femoral and tibial components. Patients were examined during standing up from and sitting down on a chair using a two-dimensional to three-dimensional registration technique.

**Results:**

During standing up from and sitting down on a chair from minimum to 30° knee flexion, anterior femoral translation was slight. From 30° knee flexion to maximum flexion, the kinematic pattern was a medial pivot and rollback.

**Conclusions:**

This study demonstrated that the knee motion kinematic patterns observed in this study were not similar to normal knee kinematics and derived from the unique design of the Bi-Surface PS.

## Background

Kinetic analysis and gait analysis are considered essential to determine the detailed effects of total knee arthroplasty (TKA). As one method for evaluating the postoperative outcome, in vivo kinematic studies are performed during knee flexion using fluoroscopy [1–6]. For the activities, knee implants partially replace the function of the lost structure’s intrinsic constraints, including the shapes of the articular surface, ligaments, and guided motion of tibial bearings. Many knee implants have been designed, such as cruciate-retaining (CR) TKA, posterior-stabilized (PS) TKA, and mobile-bearing TKA. The Bi-surface PS (Kyocera Medical Corporation, Japan) has a characteristic ball and socket joint structure in the mid-posterior portion of the femoral and tibial components. The articular surface of the tibial plate is asymmetric; it is dish-shaped on the medial side and flat on the lateral side. The post-cam mechanism is designed to start to function from 45° to 60°, which allows the femoral component to rollback early. Ball and socket joint is designed to function as the main load supporting surface from 90° and gain the anteroposterior stability.

**Fig. 1:**
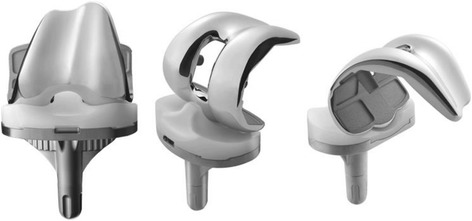
Bi-surface PS type. The characteristic ball and socket joint structure in the mid-posterior portion of the femoral and tibial components

Design of knee implants is very important and seems to be related to outcome of TKA. Thus, knee prostheses need to have even more superior performance and stability during daily living. Hence, it is necessary to understand the relationship between implant design and functional knee motion during these activities. Among daily activities, standing up from and sitting down on a chair and stair stepping are very important in daily living. Previously, we reported in vivo kinematics of this implant during a stepping exercise [7]. The goal of this analysis was to assess in vivo knee motion during standing up from and sitting down on a chair motion pattern in patients with the Bi-Surface PS functions (Fig. 1).

Three-dimensional (3D) positions and orientations of the implant components were determined using a two-dimensional to three-dimensional (2D/3D) registration technique involving previously reported techniques, manual matching, and image space optimization routines [1–3, 8]. Using this system, we performed an in vivo kinematic analysis of standing up from and sitting down on a chair in patients implanted with the Bi-Surface PS knee system. We hypothesized that the kinematics of Bi-Surface PS functions as designed during standing up from and sitting down on a chair and is similar to normal knee kinematics.

## Methods

Fifteen subjects that had undergone TKA with a Bi-Surface PS knee prosthesis (Kyocera Medical Corporation, Japan) were assessed in this study. The patients had undergone clinically successful TKA and were willing to participate in this study. All of the patients were followed-up for more than 6 months before being assessed. There were 12 female and 3 male patients, all of whom had been diagnosed with osteoarthritis. The subjects’ mean age was 72.7 ± 6.8 years (range 59–83). One surgeon performed all of the TKA procedures, and a parapatellar approach was used in all cases. The patella was not resurfaced. All of the implants were fixed in place with cement. At the time of the analysis, the mean duration of the postoperative follow-up period was 7.1 ± 1.2 months (range 6–11 months). Clinical evaluations were performed according to the knee-rating scale of the Hospital for Special Surgery (HSS) after the TKA. The patients’ mean postoperative HSS score was 91.9 ± 3.3 (range 86–97) (Table 1).

**Table 1:** Patient characteristics

Mean age	72.7 ± 6.8
Gender (male/female)	3/12
Mean body mass index	22 ± 3.6
Diagnosis (OA)	15
HSS score	91.9 ± 3.3
Mean follow-up (months)	7.1 ± 1.2

The patients were examined while standing up from and sitting down on a chair of 45 cm in height under fluoroscopic surveillance in the sagittal plane. Their foot position was determined in a neutral rotation so that they could stand up from and sit down on a chair in 10 s.

**Fig. 2:**
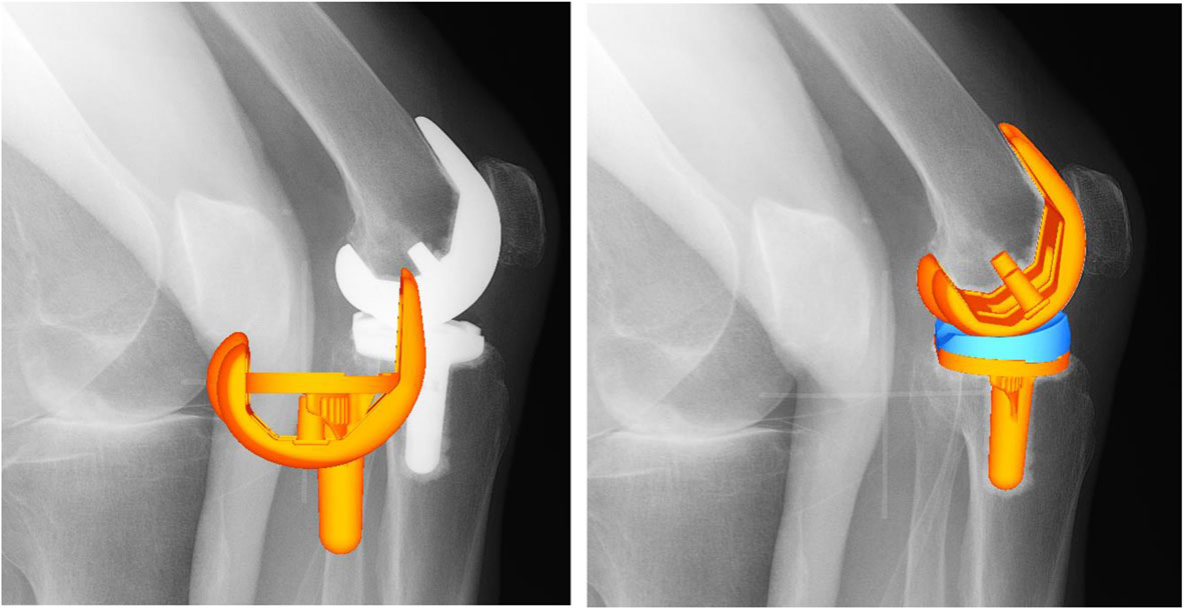
2D/3D registration technique. In vivo three-dimensional positions and orientations of the femoral component and tibial component using 2D/3D registration technique

Three measurements were recorded, and the best recording was used for the analysis. Successive knee motions were recorded as serial digital X-ray images (2048 × 1536 × 14 bits/pixels, 194-μm serial spot images as DICOM files) using a 40 cm × 30 cm flat panel detector system (DHF-155H3, Hitachi, Japan) and 1.2 to 2.0 ms pulsed X-ray beams. Three-dimensional in vivo images of the Bi-Surface PS prosthesis were created at intervals of 5° flexion using a 2D/3D registration technique. Digital fluoroscopic images were undistorted using a custom MATLAB program. The optical geometry of the fluoroscopic system (principal distance, principal point) was determined from images of a calibration target [3, 8]. An implant surface model was projected onto the geometry-corrected fluoroscopic images, and its 3D position was iteratively adjusted to match its silhouette with the silhouette of the subject’s TKA components using custom software (JointTrack, University of Florida, FL) (Fig. 2). After the matching procedure, videos of the movement of TKA components were created and subjected to a quantitative examination, and the 6 degrees of freedom kinematics of the implant’s components were calculated and subjected to quantitative analysis (3D-JointManager, GLAB Inc., Hiroshima, Japan). Data obtained via this shape-matching process have standard errors of approximately 0.53 mm for in-plane translation, 1.6 mm for out-plane translation, and 0.54° for rotation [9]. The relative movements of the femoral and tibial components were determined from the 3D positions of each component using the projection coordinate system proposed by Andriacchi [5].

We evaluated flexion angle, axial rotation angle, anteroposterior translation, valgus/varus angle, and post-cam engagement between the femoral and tibial components during standing up from and sitting down on a chair. In fixed-bearing TKA, the 3D position of the radiolucent tibial polyethylene insert was determined from the estimated position of the tibial component. The anteroposterior translation of the points on the medial and lateral sides at which the distance between the femoral component and the tibial polyethylene insert was shortest was also evaluated. External axial femoral rotation was considered to be positive, and internal axial femoral rotation was defined as negative. For each of the medial and lateral sides, the point at which the distance between the femoral component and the tibial polyethylene insert was shortest was determined by calculating the distance between the surfaces of these components using computer-aided design models. Anteroposterior locations were defined as the distance between each condylar lowest point and the anteroposterior center of the tibial baseplate. Positive value means anterior to the centerline of the baseplate (Table 2). The valgus/varus angles (varus angles are positive) of the implant were also evaluated. We defined post-cam engagement as when the nearest point between the post and cam was less than 1 mm. All data are expressed as mean ± SD values. Welch’s *t* test was used for comparisons between the anteroposterior displacement of the medial and lateral condyles or valgus/varus angles. *P* values of <0.05 were considered statistically significant.

**Table 2:** Positions of femoral component relative to tibial insert

	Positive	Negative
Rotation	External	Internal
AP translation	Anterior	Posterior

## Results

The minimum flexion angles between the femoral and tibial components were −1.7 ± 5.9° during standing and sitting; the maximum flexion angle was 78.3 ± 8.6° during standing and 78.7 ± 8.8° during sitting.

**Fig. 3:**
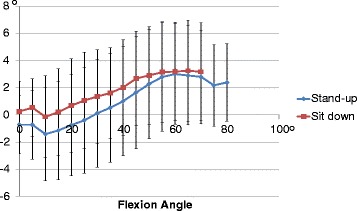
Axial rotation (mean ± SD) during standing up from and sitting down on a chair. The mean axial rotation of the femoral component exhibited gradual external rotation from 10° knee flexion to 60° knee flexion

The axial rotation angles of the femoral component relative to the tibial components were 4.0 ± 3.6° during standing and 3.1 ± 3.8° during sitting. During standing and sitting, the mean axial rotation of the femoral component exhibited gradual internal rotation from minimum knee flexion to 10° knee flexion. The mean axial rotation of the femoral component exhibited gradual external rotation from 10° knee flexion to 60° knee flexion (Fig. 3).

**Fig. 4:**
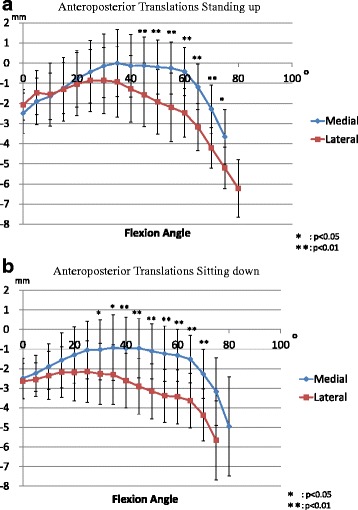
**a**, **b** Anteroposterior translations (mean ± SD) of the medial and lateral condyle nearest points during standing up from and sitting down on a chair. The level of medial anteroposterior translation was 4.1 ± 1.6 mm during standing and 3.4 ± 2.1 mm during sitting. The level of lateral anteroposterior translation was 5.8 ± 2.8 mm during standing and 4.2 ± 2.5 mm during sitting

**Fig. 5:**
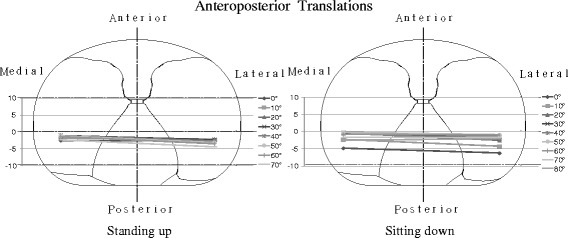
Patterns of kinematic pathways. From minimum to 30° knee flexion, anterior femoral translation was slight. From 30° knee flexion to 60° flexion, kinematic pattern was a medial pivot. From 60° knee flexion to maximum flexion, the kinematics changed into bicondylar rollback, which both condyles moved backward

From minimum flexion to maximum flexion, the level of medial anteroposterior translation was 4.1 ± 1.6 mm during standing and 3.4 ± 2.1 mm during sitting. The level of lateral anteroposterior translation was 5.8 ± 2.8 mm during standing and 4.2 ± 2.5 mm during sitting (Fig. 4a, b). From the positions of the medial and lateral femoral condyles at each flexion angle, patterns of kinematic pathways were determined. From minimum to 30° knee flexion, anterior femoral translation was slight. From 30° knee flexion to 60° flexion, kinematic pattern was a medial pivot. From 60° knee flexion to maximum flexion, the kinematics changed into bicondylar rollback, which both condyles moved backward (Fig. 5).

**Fig. 6:**
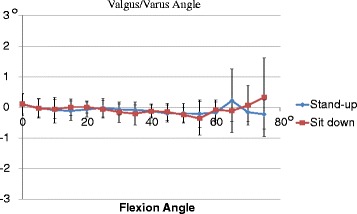
Valgus/varus angles (mean ± SD) during standing up and sitting down. No significant differences in valgus/varus angles were observed between the groups

Total valgus/varus angles for each knee were −0.1 ± 0.2° during standing and −0.1 ± 0.2° during sitting (Fig. 6). No significant differences in valgus/varus angles were observed between the groups.

Post-cam engagement was considered to have occurred in 12 cases during standing and 9 cases during sitting. The minimum flexion angle was 56.6° during standing and 59.6° during sitting.

## Discussion

Knee prostheses need to exhibit good performance and stability during daily activities. However, they do not necessarily restore normal joint stability and motion. In normal knee joints, the femur exhibits a medial pivot motion relative to the tibia during deep knee flexion. Lateral femoral condyle moved anteroposteriorly more than medial femoral condyle, and rollback does not occur medially [10–14]. On the other hand, while the center of rotation is predominantly on the lateral side of the knee during walking, the normal function of the normal knee during walking is associated with lateral and medial pivoting [15]. It has been reported that medial pivot shifts do not occur in all patients that undergo TKA [16–21]. Dennis observed both medial pivot and lateral pivot kinematic patterns in patients that had undergone TKA [16]. Banks and Hodge found that in patients that undergo successful TKA, knee motion is directly related to the constraints of the implant [22]. We reported in vivo kinematics of this implant during stair stepping exercise. It became clear that the joint’s stability during stair-stepping was affected by the design of the femorotibial joint rather than the post-cam engagement or ball and socket joint [7].

During standing up from and sitting down on a chair motion, slight anterior femoral translation occurred during the transition from minimum to 30° knee flexion; a medial pivot kinematic pattern was observed from 30° to 60° knee flexion; and a bicondylar rollback kinematic pattern, in which both condyles moved parallely backward, was seen from 60° knee flexion to maximum knee flexion. This motion patterns are different from normal knee motion [10–14] and are probably due to the post-cam engagement. This kinematic pattern was observed during standing motion greater than during sitting motion. In these exercises, slight anterior femoral translation occurred between minimum and shallow knee flexion. Post-TKA knee joint stability between minimum and shallow knee flexion was good. We considered that the main reason for the abovementioned kinematic differences was this unique design of the Bi-Surface PS.

The post-cam mechanism in the Bi-Surface PS type implant is designed to start to function from 45° to 60° of knee flexion, and the ball and socket joint functions as the main load supporting surface from 90°. During standing up from and sitting down on a chair, it was considered that post-cam engagement and ball and socket joint function occurred during standing in 12 cases and during sitting in 9 cases. The minimum flexion angle was 56.6° during standing and 59.6° during sitting. In our previous report concerning stair stepping motion in the Bi-Surface PS, post-cam engagement was considered to have occurred during step-up in one case. The minimum flexion angle during step-up was 55.1°. Furthermore, the ball and socket joint did not function in any case [7]. Thus, post-cam engagement and ball and socket joint function played a greater role as knee flexion increased. The joint stability of Bi-Surface PS type implants during shallow knee flexion is affected by the design of the femorotibial joint rather than by post-cam engagement or the functions of the ball and socket joint. However, post-cam engagement was not seen during standing and sitting motion in every case. Post-TKA knee joint stability is not only affected by the design of the implant but also by various factors such as muscular strength, ligament balance, and component positioning. We consider that the kinematic patterns exhibited by knee prostheses affect the long-term outcomes of TKA. The relationship between these kinematic patterns and clinical results should be assessed in further studies involving long-term follow-up.

There were some limitations in our study. The small number of patients weakens the statistical power of the results. Further investigation in a larger sample size and longer follow-up time is needed to obtain more overall clinical data. In addition, no control group and high standard error of measurement may have decreased the generalization power of this study. Despite all these limitations, the present study contributes significantly to the improvement of design of knee prosthesis.

## Conclusions

The kinematic pattern during standing up from and sitting down on a chair, from minimum to 30° knee flexion, anterior femoral translation was slight. From 30° knee flexion to 60° flexion, kinematic pattern was a medial pivot. From 60° knee flexion to maximum flexion, the kinematic pattern bicondylar rollback in which both condyles moved parallely backward. We consider that the knee motion kinematic patterns observed in this study were not similar to normal knee kinematics and derived from the unique design of the Bi-Surface PS.

## Abbreviations

2D/3D: Two-dimensional to three-dimensional
3D: Three-dimensional
CR: Cruciate-retaining
HSS: Hospital for Special Surgery
PS: Posterior-stabilized
SD: Standard deviation
TKA: Total knee arthroplasty
